# Supplementary material for: Bioengineered Carboxymethylcellulose–Peptide Hybrid Nanozyme Cascade for Targeted Intracellular Biocatalytic–Magnetothermal Therapy of Brain Cancer Cells
Source: Pharmaceutics. 2022 Oct 18;14(10):2223. doi: 10.3390/pharmaceutics14102223 (PMC9611945; doi:10.3390/pharmaceutics14102223)
Supplement: Supplementary file 1 [file pharmaceutics-14-02223-s001.zip › pharmaceutics-1925454-supplementary.pdf]

Supplementary Materials

Bioengineered Carboxymethylcellulose–Peptide Hybrid Nanozyme Cascade for Targeted Intracellular Biocatalytic–Magnetothermal Therapy of Brain Cancer Cells

Alexandra A. P. Mansur[1], Sandhra M. Carvalho[1], Luiz Carlos A. Oliveira[2], Elaine Maria Souza-Fagundes[3], Zelia I.P. Lobato[4], Maria F. Leite[3], Herman S. Mansur[1,*]

[1]Center of Nanoscience, Nanotechnology, and Innovation - CeNano[2]I, Department of Metallurgical and Materials Engineering, Federal University of Minas Gerais – UFMG, Av. Antônio Carlos, 6627 – Engineering School, 31.270-901, Belo Horizonte/MG, Brazil;

[2]Departament of Chemistry, Federal University of Minas Gerais – UFMG, Brazil.

[3]Department of Physiology and Biophysics, Institute of Biological Sciences-ICB, Federal University of Minas Gerais – UFMG, Brazil.

[4]Veterinary School, Federal University of Minas Gerais – UFMG, Brazil.

*e-mail: hmansur@demet.ufmg.br

Experimental Procedure

Materials

Sodium carboxymethyl cellulose (CMC, degree of substitution 0.77 and average molar mass 90 kDa), ferric chloride hexahydrate, ferrous sulfate heptahydrate, cobaltous acetate tetrahydrate, ammonium hydroxide, trisodium citrate dehydrate (TSC), tetrachloroauric(III) acid trihydrate, citric acid, sodium phosphate dibasic, horseradish peroxidase (HRP), 3,3',5,5' tetramethylbenzidine (TMB), β-D-glucose, 3-[3-dimethylaminopropyl]carbodiimide hydrochloride (EDC), and N-hydroxysulfosuccinimide (sulfo-NHS) were supplied by Sigma-Aldrich, (USA). Hydrogen peroxide was provided by Merck (Germany). Targeting peptide iRGD was supplied by Genscript Biotech Corp. The chemicals mentioned above were used without further purifications, and deionized water (DI water, Millipore Simplicity™) with resistivity ≥ 18 MΩ.cm was used to prepare the solutions. The

* To whom correspondence should be addressed: Federal University of Minas Gerais, Av. Antônio Carlos, 6627 – Escola de Engenharia, Bloco 2 – Sala 2233, 31.,270-901, Belo Horizonte/MG, Brazil; TelFax: +55-31-34091843; E-mail: hmansur@demet.ufmg.br (H. Mansur)

protocols and procedures were performed at room temperature (RT, 25 ± 2 $^{\circ}$C) unless otherwise specified.

The chemicals used for biological tests were identified in the respective protocols.

Characterization of nanostructures

Fourier-transform infrared (FTIR) spectra were obtained using attenuated total reflectance (ATR, 4000 - 650 cm^{-1} using 32 scans and a 4 cm^{-1} resolution - Nicolet 6700, Thermo Fisher, USA) with background subtraction. Samples were prepared by dropping the nanozyme in an aluminum or plastic disk and drying in a hot-air oven at 40 ± 1 ˚C. All FTIR experiments were conducted in triplicate (n = 3).

X-ray photoelectron spectroscopy (XPS) analysis was performed using Mg-Kα as the excitation source (Amicus spectrometer, Kratos, Japan). All peak positions were corrected based on C 1s binding energy (284.6 eV). Samples were prepared similarly to the FTIR assay.

UV–Vis spectroscopy measurements of suspensions were conducted using a Lambda EZ-210 (PerkinElmer, USA) in transmission mode with samples in a quartz cuvette over a wavelength range between 700 and 400 nm. Individual components were tested at the same concentration of species (Co-MION and AuNPs) in the dual-nanozyme conjugates.

The quantitative chemical analysis of Co-MION@CMC was performed to evaluate cobalt doping using wavelength-dispersive X-ray fluorescence spectrometry (WD-XRF, PW 2400 spectrometer, PHILIPS, Almelo, the Netherlands), where the concentration was determined from the Co K_{α} line intensity.

Morphological characterization was performed using a Tecnai G2-20-FEI (FEI Company, USA) transmission electron microscope (TEM) at an accelerating voltage of 200 kV coupled with energy-dispersive X-ray spectroscopy (EDS, EDAX detector coupled to Tecnai G2- 20-FEI) for the evaluation of elemental composition. In all of the TEM analyses, the samples were prepared by dropping diluted colloidal suspensions onto a porous carbon copper grid and drying at room temperature.

Dynamic light scattering (DLS) and zeta potential analyses were performed using a ZetaPlus instrument (Brookhaven Instruments Corporation, USA) with a laser wavelength of 660 nm, angle of 90°, and at RT (35 mW red diode laser), using a minimum of 10 replicates.

The magnetic properties of Co-MION@CMC were assessed by vibrational sample magnetometry (VSM) and magnetic hyperthermia experiments. The magnetization versus magnetic field for Co-MION@CMC nanoparticles at room temperature M(H) was measured in the field range from −15000 Oe to 15000 Oe at 301 K using a vibrating-sample magnetometer (VSM - model 7404, Lake Shore, USA). Magnetic hyperthermia experiments were performed using a Magnetherm^{TM} instrument (Staffordshire, UK). The Co-MION@CMC suspension (2.5 mg/mL) was exposed to an alternating magnetic field (AMF) with an amplitude of 19.9 kA/m (250 Oe) and a frequency of 112.6 kHz for 30 min. The relative temperature increase values (ΔT) of the Co-MION nanoconjugates relative to the control sample (water) were determined by subtracting the initial temperature from the temperature measured at each timepoint. The specific absorption rate (SAR, W/g_{metal}) was estimated from the initial slope of the ΔT x time curve to consider the criterion of (quasi-)adiabatic conditions (linear fitting, $R^2 \geq 0.9999$) according to Eq. (S1).

$$SAR = \frac{C\rho}{\phi}\frac{dT}{dt} \qquad (S1)$$

where C is the specific heat of the colloid, ρ is the density of the colloid, φ is the concentration of metal per mL of solution, and dT/dt represents the initial slope of the time-dependent temperature curve.

The catalytic activity of nanoconjugates—in vitro acellular analysis

Concentrations of nanoformulations:

The nanoformulations were designed based on previously published papers from our group [20,26], considering the concentrations of AuNPs and Co-MION (10 mol% of Fe^{2+} replaced by Co^{2+}) associated with cell viability/toxicity in vitro, as presented in Table S1.

Table S1. Cytotoxicity of the nanostructures and nanoconjugates at concentrations designed for this work, supported by previous reports of our group [20,26].

Sample	AuNPs	Co-MION
AuNPs – 0.35 μg/mL	Non-cytotoxic	-
AuNPs – 3.5 μg/mL	Non-cytotoxic	-
AuNPs – 35 μg/mL	Cytotoxic	-
Co-MION – 1.5 μg/mL	-	Non-cytotoxic
Co-MION – 15 μg/mL	-	Cytotoxic
Co-MION – 150 μg/mL	-	Cytotoxic
AuNP//Co-MION - 0.35 μg/mL//1.5 μg/mL	Non-cytotoxic	Non-cytotoxic
AuNP//Co-MION - 3.5 μg/mL//15 μg/mL	Non-cytotoxic	Cytotoxic
AuNP//Co-MION - 35 μg/mL//150 μg/mL	Cytotoxic	Cytotoxic

Activity of Co-MION@CMC nanozymes: The assay of peroxidase (POD) activity was prepared by adding 15 μL of Co-MION@CMC or Co-MION@CMC//AuNP suspension (Co-MION = 300 μg/mL and AuNPs = 67 μg/mL) and 20 μL of TMB solution (1 mg/mL in DI water) to a microplate well. In the sequence, citrate–phosphate buffer (pH 5.0 ± 0.2 and 7.0 ± 0.2) and H_2O_2 solution (stock solution = 0.2 M in DI water) were added in this order to reach the desired concentration of substrate (0, 5.0, or 50.0 mM) in each well (V_{total} = 200 μL). The TMB oxidized species (TMB_{ox}) were detected by absorption measurements (iMark™ Microplate Absorbance Reader, Bio-Rad®) with a wavelength filter at λ = 655 nm. The results were presented as the mean and standard deviation (SD) of eight replicates (n = 8). The effects of temperature (25 °C and 40 °C; pH = 3.0 ± 0.2; 22.5 μg/mL of nanozyme; 0.1 mg/mL of TMB; 10 mM of H_2O_2) and nanozyme concentration (2.25 μg/mL and 22.5 μg/mL; pH = 5.0± 0.2; 25 °C; 0.1 mg/mL of TMB; 50 mM of H_2O_2) were also evaluated. These tests were performed in cuvettes (V_{total} = 2.0 mL), and TMB_{ox} species were detected at 645 nm based on UV–Vis measurements (Lambda EZ-210, PerkinElmer). Data were presented as the mean and standard deviation (SD) of two replicates (n = 2).

Activity of AuNP nanozymes: The glucose oxidase (GOD) activity of AuNPs was assayed as follows: First, 500 μL of AuNP suspension (20 μg/mL) was centrifuged (12,500 rpm for 10 min), the supernatant was removed, and the AuNPs were resuspended in 500 μL of citrate–phosphate buffer (pH 5.0 ± 0.2 and 7.0 ± 0.2). Then, 250 μL of β-D-glucose solution (stock solution = 1.2 M in different buffers) was added to the gold nanoparticle suspension. After an incubation time of 60 min in the

dark, the AuNP suspension was centrifuged. Next, the generation of H_2O_2 in the supernatant was evaluated 5 min after adding 5.5 µL of HRP solution (0.1 mg/mL in DI water) and 61 µL of TMB solution (1 mg/mL in DI water) to 200 µL of the supernatant (V_{total} = 266.5 µL).

For evaluation of the oxidase-like behavior of AuNPs in the dual-nanozyme system, 150 µL of Bi-nano suspension (Co-MION = 300 µg/mL and AuNP = 67 µg/mL) was added to 350 µL of citrate–phosphate buffer at the desired pH. Then, 250 µL of β-D-glucose (stock solution = 1.2 M in different buffers) was injected and incubated in the dark for 30 min. In the sequence, the sample was centrifuged, and H_2O_2 was detected as described in the previous paragraph using HRP and TMB.

The activity of inorganic dual-nanozyme cascade: The catalytic activity of the Co-MION@CMC//AuNP (Bi-nano) cascade was tested by sensing the β-D-glucose substrate via the oxidation of TMB mediated by endogenous H_2O_2. The TMB_{ox} species were detected by UV–Vis measurements (Lambda EZ-210, PerkinElmer) at a wavelength of λ = 645 nm. The cascade activity was performed by adding 150 µL of Bi-nano suspension (Co-MION = 300 µg/mL and AuNP = 67 µg/mL), 954.2 µL of citrate–phosphate buffer at pH 5.0 ± 0.2 and 7.0 ± 0.2, 667 µL of β-D-glucose solution (stock solution = 1.2 M in citrate–phosphate buffer at the different pH values; final concentration in cuvettes of 400 mM), and 228.8 µL of TMB solution (1 mg/mL in DI water).

Biocatalytic and Magnetic hyperthermia assays in vitro with live cell cultures

All biological tests were conducted according to ISO 10993-5:2009/(R)2014 (Biological evaluation of medical devices: Tests for in vitro cytotoxicity). HEK 293T (passage 11) and U87 (passage 44) cells were cultured in DMEM (Dulbecco's modified Eagle medium, Gibco BRL, USA) with 10% FBS (fetal bovine serum, Cripion Biotecnologia Ltda., Brazil), penicillin G sodium (10 units mL^{-1}, Gibco BRL, USA), streptomycin sulfate (10 mg mL^{-1}, Gibco BRL, USA), and amphotericin B (0.025 mg mL^{-1}, Gibco BRL, USA) in a humidified atmosphere of 5% CO_2 at 37 °C.

Human embryonic kidney cells (HEK 293T, American Type Culture Collection - ATCC® CRL 1573) were provided by the Federal University of Minas Gerais (UFMG). Human brain likely glioblastoma cells (U87, ATCC® HTB-14™) were purchased from the Brazilian Cell Repository (Banco de Células do Rio de Janeiro: BCRJ, Brazil; cell line authentication molecular technique, *Short Tandem Repeat (STR) DNA*; quality assurance based on the international standard NBR ISO/IEC 17025:2005).

Statistical significance was tested using one-way ANOVA followed by Bonferroni's test. An α confidence level < 0.05 was considered statistically significant.

Measurement of intracellular ROS: U87 and HEK 293T cells (1×10^4 cells/well on 96-well plates) were incubated with 100 µL of 2',7'-dichlorodihydrofluorescein diacetate (DCF-DA, Sigma-Aldrich, USA) solution at 100 µM (diluted in DMEM medium) for 40 min in an oven at 37 ˚C in an atmosphere of 5% CO_2. After this period, the probe was removed, and the U87 cells were exposed to 100 µL of the following samples: Co-MION@CMC at 1.5, 15, and 150 µg/mL; AuNPs at 0.35, 3.5, and 35 µg/mL; and Co-MION@CMC//AuNP at 0.35//1.5, 3.5//15, and 35//150 µg/mL. HEK 293T cells were exposed to Co-MION@CMC//AuNP at 3.5//15 µg/mL. Cells were incubated for the negative control sample with DCF-DA, and positive control cells were treated with tert-butyl hydrogen peroxide (TBHP, Sigma-Aldrich, USA, 5.0 µM in water). After incubation times of 15 min, 30 min, 60 min, and 120 min, at 37 ˚C/5 % CO_2, the fluorescence intensity of DCF was measured using a Varioskan™ LUX multimode microplate reader (Thermo Scientific, $\lambda_{excitation}$ = 488 nm, and $\lambda_{emission}$ = 528 nm). When comparing cells, the PL intensity values were expressed as a percentage of fluorescence intensity relative to the positive control wells (100%). Data were presented as the mean and standard deviation of three replicates (n = 3).

MTT protocol: All cells were plated (1×10^5 cells/well) in 96-well plates. Cell populations were synchronized by nutrient deprivation for 24 h (DMEM medium without FBS). After that, the medium volume was suctioned, replaced with DMEM containing 10% FBS, and incubated for 24 h. Then, the nanozyme suspension solutions were added to individual wells at each final concentration per well as follows: Co-MION@CMC at 1.5, 15, and 150 µg/mL; AuNPs at 0.35, 3.5, and 35 µg/mL; and Co-MION@CMC//AuNP at 0.35//1.5, 3.5//15, and 35//150 µg/mL. For tests with HEK 293T cells and iRGD studies, samples of Co-MION@CMC//AuNP were added at a final concentration of 3.5//15 µg/mL. Control samples were designed as follows: control group (cell culture with DMEM and 10% FBS); positive control (cell culture with DMEM, 10% FBS, and 1.0% v/v Triton™ X-100, Sigma-Aldrich, USA); and negative control (cell culture with DMEM, 10% FBS and chips of sterile polypropylene Eppendorf®, 1 mg/mL, Eppendorf, Germany). After 24 h, the total volume of the solution in each well was aspirated and replaced with 60 µL of culture medium containing serum. The MTT reagent (5 mg/mL, >98%, Sigma-Aldrich, USA) was added to each well and incubated for

4 h in an oven at 37 ˚C in an atmosphere of 5% CO_2. Next, 40 μL of SDS (sodium dodecyl sulfate, ≥99.0%, LCG-Biotecnologia, Brazil) solution/4% HCl (37%, Sigma-Aldrich, USA) was placed in each well and incubated for 16 h in an oven at 37 ˚C in an atmosphere of 5% CO_2. Then, a volume of 100 μL from each well was aspirated and transferred to a similar blank 96-well plate, and the absorbance was measured using the iMark™ Microplate Absorbance Reader (Bio-Rad®) with a wavelength filter at λ = 595 nm. The percentage of cell viability was calculated after blank corrections according to Eq. (S2), where the values of the control group were set to 100% cell viability. Data were presented as the mean and SD of six replicates (n = 6).

$$Cell\ viability = \frac{Absorbance\ of\ sample\ and\ cells}{Absorbance\ of\ control} \text{ x } 100 \qquad \text{(S2)}$$

Determination of lipid peroxidation: U87 cells were plated (2×10^4 cells/well) in 6-well plates. Then, the nanozyme suspension solutions were added to individual wells at final concentrations per well as follows: Co-MION@CMC at 1.5, 15, and 150 µg/mL; AuNPs at 0.35, 3.5, and 35 µg/mL; and Co-MION@CMC//AuNP at 0.35//1.5, 3.5//15, and 35//150 µg/mL. Control samples were designed as follows: control group (cell culture with DMEM and 10% FBS); positive control (cell culture with DMEM, 10% FBS and 5 µM of TBHP); and negative control (cell culture with DMEM, 10% FBS, and chips of sterile polypropylene Eppendorf®, 1 mg/mL). After 24 h of incubation, cells were washed twice with Tris–HCl buffer (400 mM, pH 7.3; Labsyntth Produtos para Labotatórios Ltda, Brazil) and treated with 1 mL of a solution containing 0.4% (w/v) thiobarbituric acid (TBA, Sigma-Aldrich, USA), 0.5% (w/v) SDS, and 5% (v/v) acetic acid (Labsyntth Produtos para Labotatórios Ltda, Brazil), pH 3.5. The cells were scraped and incubated in the sequence at 95 °C (Termomix, Eppendorf F1.5). After 60 minutes, the reaction was stopped in an ice bath for 5 minutes. Then, a volume of 300 µL was transferred to a blank 96-well plate, and the absorbance was measured using a Varioskan™ LUX multimode microplate reader (Thermo Scientific) at λ = 532 nm. The results were calculated as nmol of MDA-TBA/mg of cellular protein, using 156 $mM^{-1}cm^{-1}$ as the molar extinction coefficient of MDA-TBA. Proteins extracted from cells were calculated using the Bradford method (Bradford reagent, Sigma-Aldrich, USA), using bovine serum albumin (BSA,

Sigma-Aldrich, USA) as a reference material. Data were presented as the mean and SD of three replicates (n = 3).

Cellular Magnetic Hyperthermia (MHT) in vitro: U87 cells were plated (1×10^5 cells/well) in 24-well plates. Cell populations were synchronized in serum-free media for 24 h. After that, the medium volume was suctioned and replaced with Leibowitz medium (L-15, Gibco BRL, USA) containing 10% FBS for 24 h. Then, the Bi-nano and Bi-nano_iRGD suspensions were added to individual wells at a final concentration of 3.5//15 μg/mL (Co-MION@CMC//AuNP). The control groups were set as follows: negative control (cell culture with L-15 and 10% FBS); negative control + MHT (cell culture with L-15 and 10 % FBS exposed to an alternating magnetic field (AMF)); and positive control (cell culture with L-15 and 10 % FBS and 1.0% v/v Triton™ X-100). For samples without MHT treatment, after 3 h of incubation in an atmosphere without CO_2 at 37 °C, cells were washed with PBS, trypsinized for 7 min (250 μL of 0.2 % trypsin, Sigma-Aldrich), centrifuged (1400 rpm for 5 min), and resuspended in 600 μL of L-15 supplemented with 10% FBS. Cancer cell death was characterized using mitochondrial activity based on the in vitro MTT bioassay. Therefore, 400 μL of the MTT reagent (5 mg/mL) was added to each well with the cells and incubated for 2.5 h at 37 °C. Formazan crystals were dissolved using 400 μL of isopropanol solution/4% HCl. Then, 100 μL of the solution was removed from each well and transferred to a 96-well plate, and the absorbance was measured (iMark™ Microplate Absorbance Reader, Bio-Rad®, with a wavelength filter at λ = 595 nm). The experiments were performed in 10 replicates (n = 10), and the cell viability response was expressed according to Equation (S2).

For cells with MHT treatment, after 3 h of incubation in an atmosphere without CO_2 at 37 °C, these systems were exposed to an alternating magnetic field (H = 19.9 kA m^{-1}, frequency = 112.6 kHz) for 60 minutes. After exposure to the AMF, the cells were treated as previously described for the MTT bioassay.

Figures

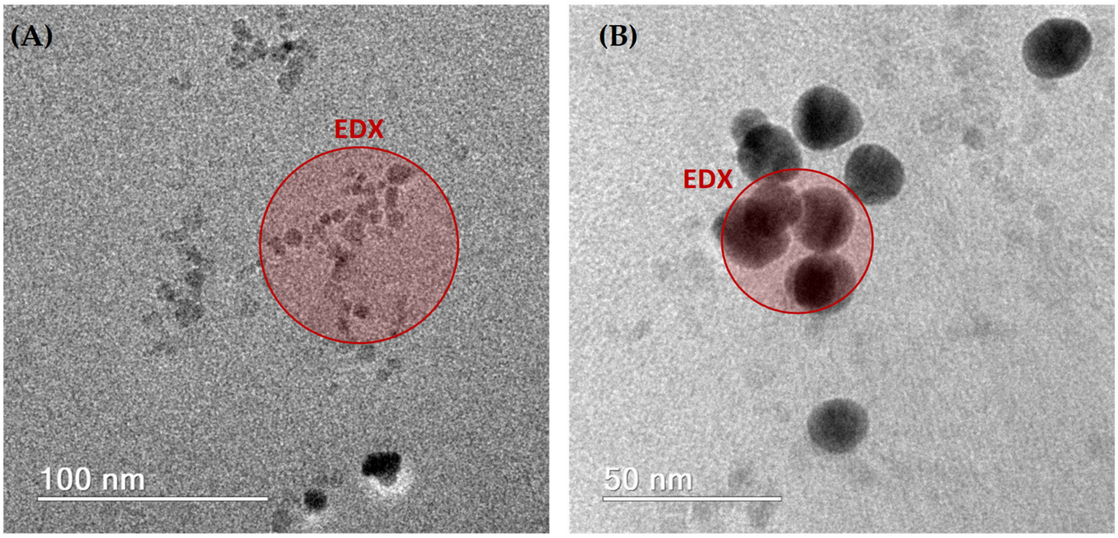

Figure S1. Selected areas in TEM images of the dual-nanozyme samples used for obtaining EDX spectra for (**A**) Co-MION and (**B**) gold (AuNP) nanozymes.

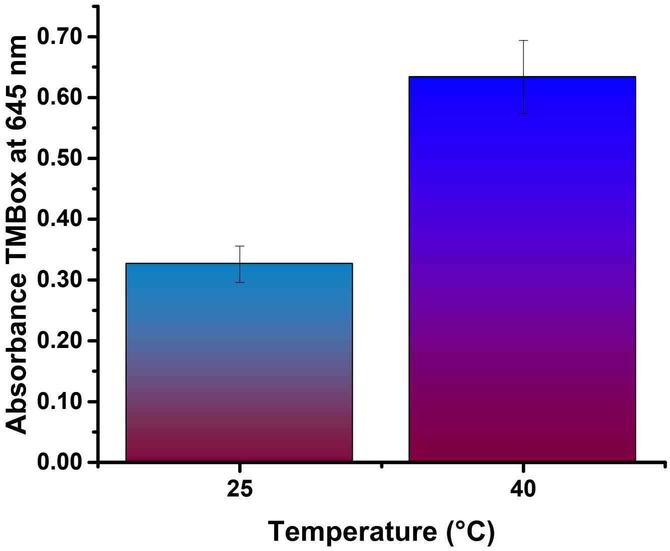

Figure S2. Evaluation of the peroxidase-like activity of Co-MION@CMC at different temperatures (after 45 min; pH = 3.0 ± 0.2; 22.5 μg/mL of Co-MION; 0.1 mg/mL of TMB; 10 mM of H_2O_2).

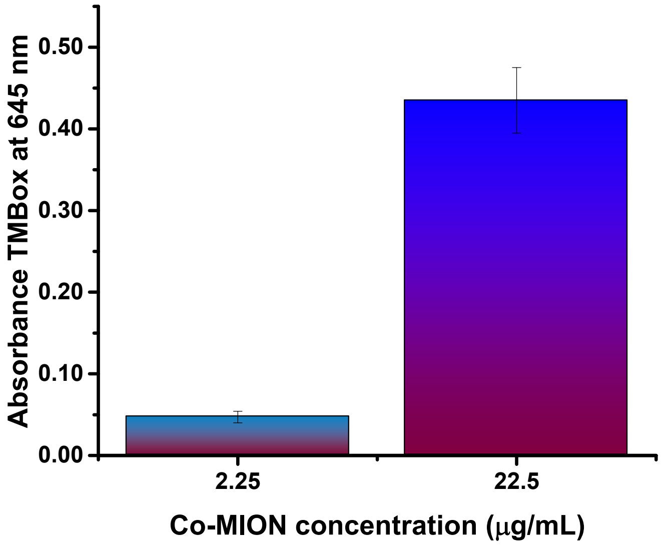

Figure S3. Evaluation of the peroxidase-like activity of Co-MION@CMC at different concentrations of nanozymes (after 30 min; pH = 5.0 ± 0.2; T = 25 °C; 0.1 mg/mL of TMB; 50 mM of H_2O_2).

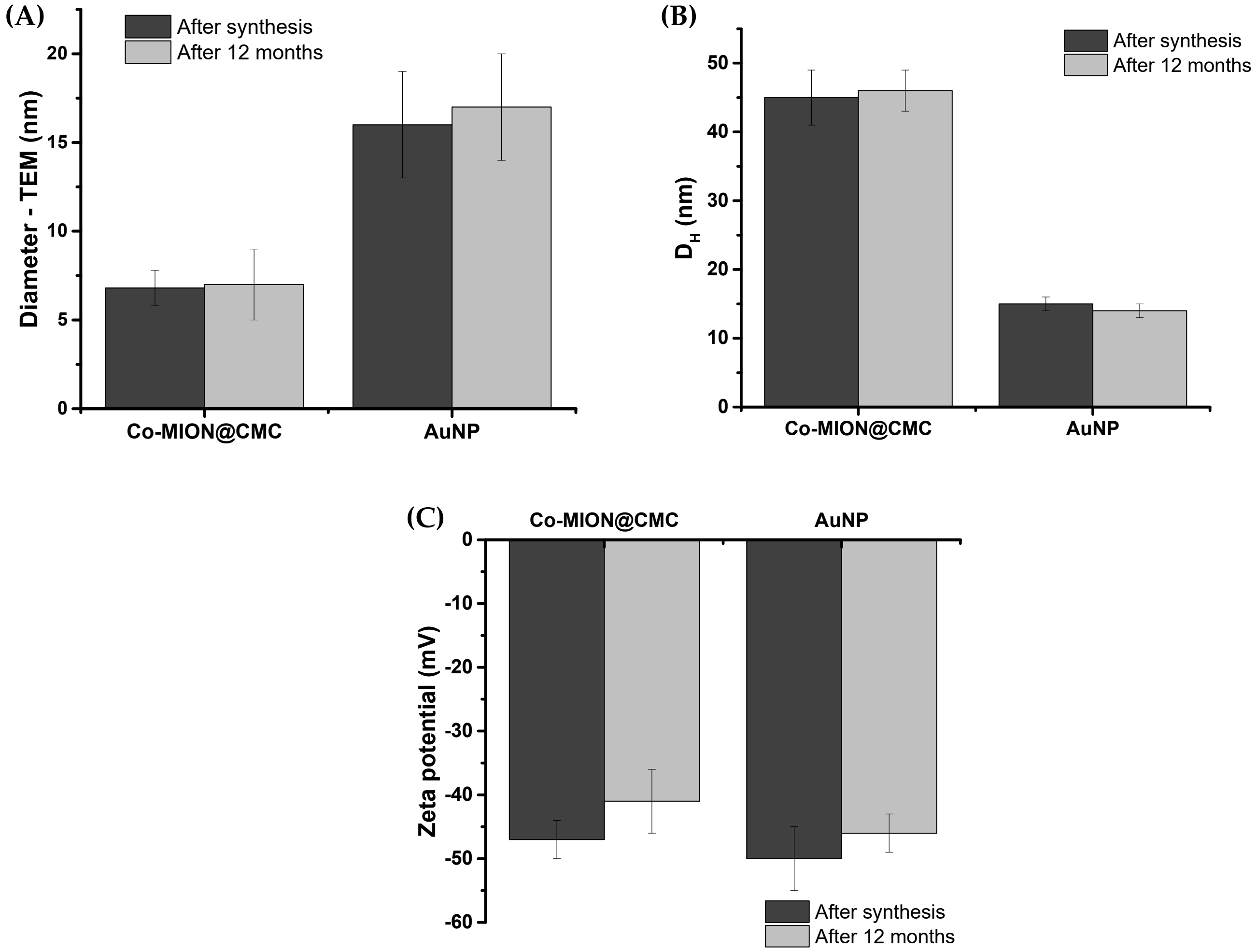

Figure S4. Co-MION@CMC and AuNP nanostructures' properties and parameters "after-synthesized" and after 12 months of storage: (**A**) Diameter (based on TEM images); (**B**) Hydrodynamic diameter; and (**C**) Zeta potential.
